# Suboptimal Responses in Chronic Myeloid Leukemia

**DOI:** 10.1002/cncr.26391

**Published:** 2011-10-28

**Authors:** Elias Jabbour, Giuseppe Saglio, Timothy P Hughes, Hagop Kantarjian

**Affiliations:** 1 Department of Leukemia, The University of Texas MD Anderson Cancer CenterHouston, Texas; 2 Division of Internal Medicine and Hematology, University of Turin and San Luigi Gonzaga HospitalOrbassano, Italy; 3 SA Pathology, University of AdelaideAdelaide, Australia

**Keywords:** tyrosine kinase inhibitor, imatinib mesylate, nilotinib, dasatinib, chronic myeloid leukemia

## Abstract

The high response rates and increased survival associated with imatinib therapy prompted a paradigm shift in the management of chronic myeloid leukemia. However, 25% to 30% of imatinib-treated patients develop drug resistance or intolerance, increasing the risk of disease progression and poor prognosis. In 2006, the European LeukemiaNet proposed criteria to identify patients with a suboptimal response to, or failure associated with, imatinib; these recommendations were updated in 2009. Suboptimal responders represent a unique treatment challenge. Although they may respond to continued imatinib therapy, their long-term outcomes may not be as favorable as those for optimally responding patients. Validation studies demonstrated that suboptimal responders are a heterogeneous group, and that the prognostic implications of suboptimal response vary by time point. There are few data derived from clinical trials to guide therapeutic decisions for these patients. Clinical trials are currently underway to assess the efficacy of newer tyrosine kinase inhibitors in this setting. Identification of suboptimal responders or patients failing treatment using hematologic, cytogenetic, and molecular techniques allows physicians to alter therapy earlier in the treatment course to improve long-term outcomes. Cancer 2012;. © 2011 American Cancer Society.

Chronic myeloid leukemia (CML) afflicts approximately 1.5 per 100,000 individuals in the United States, with an estimated 5000 new cases diagnosed annually.^1^,^2^ The etiology of CML is a chromosomal aberration known as the Philadelphia (Ph) chromosome, which is created by the reciprocal translocation of chromosomes 9 and 22 (t[9;22][q34;q11]).^3^ The Ph chromosome leads to the expression of BCR-ABL tyrosine kinase, an oncogenic fusion protein. BCR-ABL, identified in 1985 as the cause of malignant transformation in Ph^+^ CML,^4^ served as the target for design of tyrosine kinase inhibitors (TKIs).

The first TKI approved by the US Food and Drug Administration (FDA) for the treatment of Ph^+^ CML in chronic phase (CML CP) was imatinib (STI571; Gleevec; Novartis, Florham Park, NJ), which quickly replaced interferon (IFN) alpha as the standard of care. Results from the 6-year follow-up study of the International Randomized Study of Interferon Plus Ara-C Versus STI571 (IRIS) demonstrated an event-free survival (EFS) rate of 83% and an estimated rate of freedom from progression (FFP) to advanced disease (accelerated phase [AP] or blast crisis [BC]) of 93%, associated with imatinib therapy^5^; results at 8 years confirmed the durability of these responses.^6^ The newer TKIs, nilotinib (Tasigna, Novartis) and dasatinib (Sprycel; Bristol-Myers Squibb, Princeton, NJ), have been commercially available for >3 years and were indicated first for patients who developed resistance or intolerance to first-line therapies, including imatinib.^7^,^8^ In June 2010 and October 2010, nilotinib and dasatinib, respectively, were FDA approved as first-line therapy for patients with newly diagnosed CML CP.^8^ The efficacy of TKIs has extended treatment goals beyond those previously attainable with IFN-based therapies to include molecular responses. Currently recognized response milestones of treatment are complete hematologic response (CHR), complete cytogenetic response, major molecular response, and complete molecular response (or undetectable *BCR-ABL* transcripts by current technology).^9^

Despite imatinib's efficacy, 24% of newly diagnosed CML CP patients treated with imatinib in the IRIS trial failed to achieve complete cytogenetic response within 18 months of treatment, and 14% discontinued treatment or crossed over to the IFN-alpha/cytarabine arm.^10^ To assist clinicians in determining when a change in therapy may be needed based on individual patient responses, the European LeukemiaNet defined treatment responses at various time points as *failure* and *suboptimal*.^11^ Patients classified as experiencing failure would likely benefit from a change in treatment. Patients experiencing suboptimal response may benefit from continued therapy; however, long-term outcomes may not be as favorable as those for patients achieving milestone responses.^11^

Treatment failures are relatively straightforward to identify and treat; the management of suboptimal responses may be more complex. There is less awareness of what constitutes suboptimal response, and physicians may elect to continue imatinib treatment for the duration of measurable response. Furthermore, there are differences between CML guidelines regarding these defined responses. The European LeukemiaNet guidelines define suboptimal response and failure; the National Comprehensive Cancer Network guidelines define target responses by specific time points. Physicians should carefully monitor each patient's response to treatment and identify patients not achieving optimal responses as early in the treatment course as possible.

## Current Monitoring Recommendations

Regularly scheduled monitoring after treatment initiation may help identify patients at risk of suboptimal response or failure. CML remission occurs in the sequence CHR, complete cytogenetic response, major molecular response, and complete molecular response. Internationally endorsed guidelines describe the expected response milestones, critical time points, and frequency of monitoring.^9^,^12^,^13^

Hematologic responses should be assessed every 2 weeks after initiating treatment, until CHR is achieved and confirmed.^9^ Bone marrow cytogenetics are recommended at diagnosis, at 3 and 6 months after treatment initiation, and then every 6 months until complete cytogenetic response is achieved and confirmed. Thereafter, cytogenetic analysis is reserved for when molecular monitoring is not feasible or for patients exhibiting myelodysplastic features (eg, cytogenetic abnormalities in diploid cells), suboptimal response, or failure.^9^ Molecular monitoring of the *BCR-ABL* transcripts by quantitative real-time polymerase chain reaction should be conducted every 3 months until a major molecular response is achieved and confirmed and at least every 6 months thereafter. Molecular monitoring has the highest sensitivity of current tests for treatment responses in CML; in clinical practice, it is typically used once patients have achieved complete cytogenetic response.^14^

## Defining Suboptimal Responses

Suboptimal responses are defined based on the lack of achievement of certain response milestones (hematologic, cytogenetic, and molecular; Table 1) at specific time points. European LeukemiaNet^9^ and the European Society for Medical Oncology^15^ have each defined suboptimal response to treatment milestones and accompanying treatment recommendations. The definitions were based on responses at time intervals observed in clinical studies, including the IRIS trial, and clinical experience.^16^ European LeukemiaNet and European Society for Medical Oncology guidelines define suboptimal response as: 1) less than a CHR at 3 months; 2) less than a partial cytogenetic response at 6 months; 3) less than a complete cytogenetic response at 12 months; 4) less than an major molecular response at 18 months; or 5) loss of major molecular response or development of partially imatinib-sensitive *BCR-ABL* mutations at any time (Table 2).^9^,^15^ The National Comprehensive Cancer Network guidelines do not define suboptimal response per se; however, treatment recommendations at specified time points after initiating TKI therapy are provided based on the response.^17^

**Table 1:** Response Criteria in Chronic Phase Ph^+^ CML According to National Comprehensive Cancer Network Guidelines^17^

Response	Criteria
**Hematologic**	
Complete	Complete normalization of peripheral blood counts (leukocyte <10 × 10^9^/L)
	Platelet count <450 × 10^9^/L
	No immature cells (eg, myelocytes, promyelocytes, or blasts) in peripheral blood
Partial	No signs or symptoms of disease; no palpable splenomegaly
	Same as complete hematologic response, except for:
	Presence of immature cells
	Platelet count <50% of pretreatment count but >450 × 10^9^/L
	Persistent splenomegaly but <50% of pretreatment enlargement
**Cytogenetic**^a^	
Complete	No Ph^+^ metaphases
Partial	1%-35% Ph^+^ metaphases
Major	0%-35% Ph^+^ metaphases (complete + partial)
Minor	>35% Ph^+^ metaphases
**Molecular**	
Complete	BCR-ABL mRNA undetectable by qRT-PCR
Major	≥3-log reduction of BCR-ABL mRNA^b^

Abbreviations: CML, chronic myeloid leukemia; Ph, Philadelphia chromosome; qRT-PCR, qualitative real-time polymerase chain reaction.

Reproduced with permission from *Annals of Internal Medicine*.

a Examination of ≥20 metaphases.

b European LeukemiaNet guidelines define major molecular response as a ratio of *BCR-ABL1* to *ABL1* or other housekeeping genes of ≤0.1% on the International Scale.

**Table 2:** ELN Criteria for Defining a Suboptimal Response^a^^9^,^25^

Testing Time	ELN Criteria^a^
3 months	No cytogenetic response
6 months	<PCyR
12 months	PCyR
18 months	<MMR
Any time	Loss of MMR, imatinib-sensitive mutations

Abbreviations: ELN, European LeukemiaNet; MMR, major molecular response; PCyR, partial cytogenetic response.

a See Table 1 for definitions of response.

## Clinical Implications of Suboptimal Response

Patients achieving optimal milestone responses have improved long-term outcomes.^5^,^18^-^24^ Patients with European LeukemiaNet-defined suboptimal response at 6 and 12 months have a worse long-term prognosis than patients with optimal responses.^25^,^26^ The predictive value of responses varies by time point (eg, responses at 3 and 6 months are qualitatively different from those at 12 and 18 months); these issues are further discussed in the subsequent section. Patients with suboptimal responses have greater risk of disease progression than optimal responders.^18^,^27^-^29^ Furthermore, patients who have transformed to AP or BC generally respond poorly to standard treatment and have higher rates of morbidity and mortality.^30^

In several clinical studies, investigators have applied the definitions of suboptimal response to treatment outcomes to determine the prognostic significance of these responses (Table 3). Data for 281 patients with CML CP participating in clinical trials at The University of Texas MD Anderson Cancer Center were retrospectively analyzed.^25^ At 6, 12, and 18 months, 4%, 8%, and 50% of patients, respectively, had suboptimal responses. The outcomes for suboptimal responders varied by time point. For patients who were suboptimal responders at 6 months, the 4-year EFS and treatment-free survival rates were significantly worse than those of optimal responders. Patients with suboptimal response at 12 months showed a trend toward lower EFS; those with suboptimal response at 18 months had a treatment-free survival comparable to optimal responders. The authors concluded that patients with suboptimal responses generally have worse long-term outcomes than patients with optimal responses, particularly if the suboptimal response occurs early in treatment.

**Table 3:** Long-Term Outcomes According to Response (Optimal vs Suboptimal^a^) to Imatinib or MMR Status at 6, 12, and 18 Months

Result	Probability of Achieving Indicated Milestones, %, MD Anderson Cancer Center^25^ (N = 281)									
Outcome	4-Year EFS		4-Year TFS		Ever Reaching CCyR		Ever Reaching MMR		Transformation	
Response	Optimal	Suboptimal	Optimal	Suboptimal	Optimal	Suboptimal	Optimal	Suboptimal	Optimal	Suboptimal
Months on treatment										
6 months	93	45	95	60	97	30	80	0	6	30
12 months	96	87	96	93	72	18	82	39	5	5
18 months	NP	NP	NP	NP	NA	NA	NA	66	4	5

	Probability of Achieving Milestone, %, MD Anderson Cancer Center^32^ (N = 258)		
Outcome	Ever Reaching CCyR	Ever Reaching MMR	Event
Response	No CCyR	No CCyR	No CCyR
Months on treatment			
6 months	57	43	34
12 months	42	31	38

	Probability of Achieving Milestone at 5 Years, %, Hammersmith Hospital UK^26^ (N = 224)							
Outcome	OS		PFS		CCyR		Loss of CCyR	
Response	Optimal	Suboptimal	Optimal	Suboptimal	Optimal	Suboptimal	Optimal	Suboptimal
Months on treatment								
6 months	96	92	91	62^b^	97	64^c^	13	11
12 months	98	85^b^	96	73^b^	100	78^c^	10	3
18 months	100	98	97	97	NA	NA	25	0

	Probability of Achieving Milestone at 5 Years, %, GIMEMA^31^ (N = 559)							
Outcome	EFS		FFS		CCyR		MMR	
Response	Optimal	Suboptimal	Optimal	Suboptimal	Optimal	Suboptimal	Optimal	Suboptimal
12 months treatment	85	42^c^	92	49^c^	98	85^c^	96	81^c^

	Probability of Achieving Milestone at 7 Years,%, IRIS^33^ (n = 476 Evaluable Patients; N = 553 Enrolled)					
Outcome	OS		EFS		Without AP/BC	
Response	MMR	No MMR	MMR	No MMR	MMR	No MMR
Months on treatment						
6 months	90	89	85	84	96	93
12 months	93	89	91	79^b^	99	90^b^
18 months	95	90	95	75^b^	99	90^b^

Abbreviations: AP/BC, accelerated phase or blast crisis; CCyR, complete cytogenetic response; EFS, event-free survival; FFS, failure-free survival; GIMEMA, Group for Hematological Malignancies of the Adult; IRIS, International Randomized Study of Interferon Plus Ara-C Versus STI571; MMR, major molecular response; NA, not applicable; NP, not provided; OS, overall survival; PFS, progression-free survival; TFS, treatment-free survival; UK, United Kingdom.

a Suboptimal response was defined as <partial cytogenetic response at 6 months, <CCyR at 12 months, and <MMR at 18 months; optimal response was defined as having a response that was greater than a suboptimal response.

b *P* ≤ .05.

c *P* < .001.

These findings are supported by an analysis of 224 patients with CML CP treated with imatinib at the Hammersmith Hospital in the United Kingdom (Table 3).^26^ In this analysis, the prognostic significance of suboptimal response also varied by time point. Compared with optimal responders, suboptimal responders at 6 and 12 months had significantly lower progression-free survival (PFS) and probability of complete cytogenetic response, and suboptimal responders at 12 months had significantly worse overall survival (OS), but suboptimal responders at 18 months did not have significantly different OS or PFS.

The Italian GIMEMA (Group for Hematological Malignancies of the Adult) CML Working Party analysis also evaluated the outcome of patients with European LeukemiaNet-defined suboptimal response (Table 3).^31^ Suboptimal responders at 6 and 12 months had significantly lower probability of achieving complete cytogenetic response, major molecular response, failure-free survival, and EFS at 5 years than optimal responders; however, OS was not different between the 2 groups.

The level of initial response also appears to predict achievement of subsequent responses. In the retrospective analysis by Alvarado and colleagues at The University of Texas MD Anderson Cancer Center, no suboptimal responders at 6 months ever achieved major molecular response, and only 30% achieved complete cytogenetic response (Table 3).^25^ Of suboptimal responders at 12 months, 39% and 72% eventually achieved major molecular response and complete cytogenetic response, respectively, resulting in a transformation rate similar to that in optimal responders but with a higher probability of an event (26% vs 8%, respectively), including loss of CHR or major cytogenetic response, progression to AP/BC, or death from any cause. Among suboptimal responders at 18 months, 66% eventually achieved major molecular response.

In another analysis, the long-term impact of delayed treatment responses was determined in 258 patients with CML CP.^32^ Patients who failed to achieve complete cytogenetic response after 3, 6, and 12 months of imatinib treatment were increasingly unlikely to achieve complete cytogenetic response (*P* = .002) or major molecular response (*P* = .004). The probability of an event—loss of CHR, loss of minor cytogenetic response, increasing white cell count, transformation to AP/BC, or death from any cause during imatinib treatment—increased as the time to complete cytogenetic response increased (*P* = .16). The authors concluded that although favorable outcomes are possible after delayed responses, patients who do not achieve early cytogenetic responses may progress before achieving optimal responses. Therefore, early identification of patients at high risk of not achieving critical milestones is crucial.^32^

Clinical trial results also suggest a benefit to achieving early major molecular response in patients receiving imatinib therapy (Table 3). A recent analysis of 7-year data by the IRIS investigators demonstrated the long-term prognostic value of molecular response at specific time points.^33^ This analysis used the complete molecular monitoring dataset, consisting of 3627 blood samples from 476 patients in the imatinib arm of the IRIS trial. *BCR-ABL* transcript levels at 6, 12, and 18 months predicted long-term EFS and FFP to AP/BC. EFS was defined as the time from treatment initiation to loss of CHR, loss of major cytogenetic response, progression to AP/BC, or death by any cause. Major molecular response by 12 or 18 months predicted EFS rates of 91% and 95%, respectively, at 7 years. However, achieving major molecular response at 12 months showed no EFS benefit when compared with not achieving major molecular response but having complete cytogenetic response; there was minimal EFS benefit when major molecular response was achieved at 18 months versus not achieving major molecular response but having complete cytogenetic response. Conversely, EFS was 79% in patients achieving major molecular response by 12 months and 75% in those not achieving major molecular response at 18 months (vs achieving major molecular response at 12 months [*P* = .001] and 18 months [*P* < .001]). When the definition of EFS was expanded to include loss of complete cytogenetic response, EFS rates were 87% for patients with major molecular response at 12 months versus 73% for patients with no major molecular response (*P* = .0006), and 92% for patients with major molecular response at 18 months versus 65% for patients with no major molecular response (*P* < .001). Major molecular response at 12 or 18 months also was associated with a significantly decreased rate of transformation to AP/BC. Estimated rates of PFS at 7-year follow-up were 99% for patients with major molecular response versus 90% for those without major molecular response at both 12 and 18 months (*P* < .001 for both comparisons).^33^ Furthermore, a reanalysis based on updated data (excluding 1 patient who died from non-CML causes) found that no patient with major molecular response at 12 or 18 months progressed to AP or BC over the 7-year follow-up. Similarly, although follow-up data in studies of nilotinib and dasatinib in a front-line setting are immature, 12-month follow-up data indicate that patients who achieved major molecular response at any time point did not transform to AP or BC.^34^

## Adherence and Suboptimal Response

**Figure 1:**
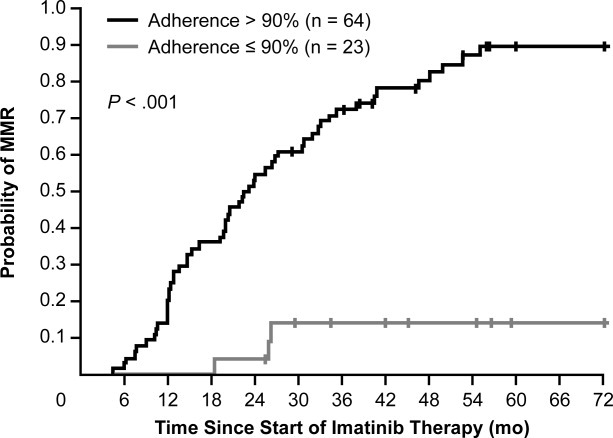
Six-year probability of major molecular response (MMR) in 87 patients treated with imatinib is shown according to the measured adherence rate. The probability of MMR for the 23 patients with an adherence rate ≤90% was 13.9%, whereas the probability of MMR for the 64 patients with an adherence rate >90% was 93.7% (*P* < .001).^35^ Reproduced with permission from *Journal of Clinical Oncology*.

Marin and colleagues from the Hammersmith Hospital have shown that the degree of patient adherence to imatinib is critical in achieving major molecular response.^35^ These investigators examined adherence patterns during a 3-month period in 87 patients with complete cytogenetic response who had been receiving imatinib for a median of approximately 5 years. Highly adherent patients (those who took >90% of medication as prescribed) had a significantly higher 6-year probability of achieving major molecular response than did less-adherent patients (94% vs 14%, respectively; *P* < .001 [Fig. .1]). Furthermore, patients who were highly adherent, even if they did not achieve major molecular response by 12 or 18 months, had a high probability of eventually achieving major molecular response.^35^ The authors performed a univariate analysis of several variables—patient age, sex, weight, plasma imatinib level, percent adherence, and Sokal risk group—and found that adherence was the only independent predictor for major molecular response. Clearly, adherence is an important parameter to investigate among patients with suboptimal response to TKI treatment.

## Assessing Adherence in Patients With Suboptimal Response

It is important that physicians maintain an index of suspicion when evaluating whether adherence is a factor in suboptimal responses in TKI-treated patients. As recommended in the National Comprehensive Cancer Network guidelines, adherence should be assessed during follow-up evaluations in any patient not achieving an anticipated response within the expected time.^17^ Although there are few studies of adherence in patients with CML, several factors have been identified as predictors of poor adherence to TKI therapy; knowledge of these factors can help physicians recognize at-risk patients.

A retrospective cohort study using a database of participants in employer-based health plans found that younger age, shorter exposure to imatinib, starting imatinib dose of ≤400 mg, longer delay between CML diagnosis and imatinib prescription fill, higher concomitant prescriptions, and higher copayment percentage were associated with poor adherence to imatinib therapy.^36^ In addition, the study by Marin and colleagues at the Hammersmith Hospital found that younger age, adverse events (AEs [specifically, asthenia, nausea, muscle cramps, and bone or joint pain]), taking imatinib independently of meals, and unexplained increases in *BCR-ABL1* transcript levels were associated with poor adherence.^35^ Several of these risk factors, particularly AEs, can be modified with prompt supportive care. Others can be addressed through patient education, improved patient-physician communication, and regular physician contact.^37^

## Treating Patients With Suboptimal Response

The optimal treatment course for patients with suboptimal response remains unclear because of a dearth of prospective randomized trials evaluating the correlation between interventions and clinical outcomes. Options, however, include increasing the dose of imatinib, changing TKI therapy to nilotinib or dasatinib, or participation in a clinical trial of an investigational agent.

### Imatinib dose escalation

Interest in optimizing the imatinib dose has arisen because the maximum tolerated dose was not determined in patients with CP; only the 400-mg/d dose was studied in these patients.^38^ Results of several studies suggest that although higher imatinib doses as first-line therapy are associated with early responses, these responses have not translated into improved EFS, PFS, or OS.^9^,^39^,^40^ In an early study of imatinib dose escalation, Kantarjian and colleagues evaluated the efficacy and safety of increasing imatinib dose to 600 or 800 mg daily in 54 patients with hematologic resistance or relapse (n = 20) or cytogenetic resistance or relapse (n = 34) on imatinib 400 mg daily.^41^ Of the 20 patients receiving escalated doses because of hematologic resistance or relapse, 65% achieved hematologic response, and 5% achieved major cytogenetic response. Of the 34 patients with cytogenetic resistance or relapse, 38% achieved major cytogenetic response with escalated imatinib doses. In a longer-term analysis of patients having a median follow-up of 61 months from dose escalation (n = 84), 40% achieved complete cytogenetic response, and 14% achieved partial cytogenetic response.^42^ Of patients who received dose escalation because of cytogenetic failure (n = 63), 52% achieved complete cytogenetic response, and 16% achieved partial cytogenetic response; of 21 patients qualifying because of hematologic failure, 5% achieved complete cytogenetic response, and 10% achieved partial cytogenetic response.

Results of a retrospective analysis of the IRIS data in patients who required imatinib dose escalation per protocol or based on European LeukemiaNet recommendations also suggest that dose escalation may benefit patients with CML CP with suboptimal cytogenetic response.^43^ The most recent data regarding imatinib dose escalation were reported in a 2010 Korean study by Koh and colleagues that included 19 patients with suboptimal response to the standard imatinib dose.^44^ Consistent with the results of the IRIS retrospective analysis, escalating the imatinib dose was found to be a reasonable option for CML patients with suboptimal response to standard imatinib doses.^44^ Response in a small number of patients receiving escalated imatinib doses was found not to be durable, generally 3 to 6 months.^45^ Collectively, these data suggest that higher imatinib doses can overcome lack of optimal response in some patients; however, higher doses may also be associated with poor tolerability.^46^

### Treatment with nilotinib in patients resistant or intolerant to imatinib

The efficacy and safety of nilotinib in patients with suboptimal response to imatinib are under investigation in several clinical trials. In the phase 3b, open-label, multicenter ENACT (Expanding Nilotinib Access in Clinical Trials) study conducted between January 2006 and October 2008, the safety and efficacy of nilotinib 400 mg twice daily were evaluated in 1422 patients with CML CP who were resistant and/or intolerant to imatinib in a clinical practice setting.^47^-^49^ Nilotinib was well tolerated overall. In a subset of patients with suboptimal cytogenetic response to imatinib (n = 12), 75%, 50%, and 67% achieved major cytogenetic response, complete cytogenetic response, and CHR, respectively, compared with 45%, 34%, and 43%, respectively, for the overall population (n = 1422).^47^ The median times to major cytogenetic response and CHR among suboptimal responders (3.8 and 3.4 months, respectively) were less than in the overall study population (6.1 and 4.9 months, respectively). On the basis of these data, nilotinib was more effective in patients with suboptimal response to imatinib at 6 or 12 months than in patients with imatinib failure.

Preliminary results of an exploratory, United States-based, multicenter, open-label study of nilotinib 300 mg twice daily in patients who achieved complete cytogenetic response but had suboptimal molecular response to imatinib (*BCR-ABL/ABL* levels >0.1% on the International Scale after ≥12 months; >1-log increase in *BCR-ABL/ABL* levels at any time) demonstrated that 6 of 8 evaluable patients (75%) achieved major molecular response after 3 months of nilotinib treatment.^50^ All patients maintained complete cytogenetic response, and 80% of evaluable patients achieved major molecular response within 9 months. An updated analysis after continued enrollment, which included 14 patients with a median of 10 months of nilotinib treatment, showed that 88% of patients achieved major molecular response at any time.^51^

Switching to nilotinib after suboptimal response or intolerance to imatinib in the TIDEL (Therapeutic Intensification in DE-novo Leukemia) II multicenter study in newly diagnosed patients with CML CP also demonstrated favorable results.^52^ In this ongoing study, patients are treated with high-dose imatinib (600 mg/d) and evaluated for imatinib intolerance or suboptimal response (as defined by *BCR-ABL* transcript levels of 10% at 3 months, 1% at 6 months, and 0.1% at 12 months by International Scale). Suboptimal responders receive a dose escalation of imatinib to 800 mg daily; if the *BCR-ABL* transcript target level is not achieved by 3 months after dose escalation or if patients become intolerant to imatinib treatment, patients are switched to nilotinib (400 mg twice daily). In an interim analysis of 105 patients with a median follow-up of 295 days, 21 patients switched to nilotinib, and 95% achieved or maintained complete cytogenetic response. With a median follow-up of 295 days after switching to nilotinib, 9 of the 12 patients who were intolerant to imatinib achieved major molecular response; 1 of the 7 patients with suboptimal response to imatinib achieved major molecular response.^52^

An ongoing open-label, randomized, phase 3 study (NCT00802841) in patients responding suboptimally to imatinib 400 mg daily is comparing imatinib dose escalation (600 mg daily) with switching to nilotinib 400 mg twice daily. Eligibility requirements include 3 to 18 months of imatinib treatment and suboptimal response to imatinib (defined as no cytogenetic response at ≥3 to <6 months of treatment, no partial cytogenetic response at ≥6 to <12 months of treatment, and no complete cytogenetic response at ≥12 to <18 months of treatment).

Additional prospective trials of nilotinib are underway. A phase 3 study comparing nilotinib 400 mg twice daily with imatinib 600 mg daily in patients with suboptimal response to imatinib 400 mg daily (LASOR) is ongoing, with primary outcome data expected in early 2014. A phase 2 single-arm study (ENABL) is evaluating nilotinib in patients with a suboptimal response to imatinib. Preliminary data for 14 patients have been presented; of 7 patients with at least 12 months of treatment, 6 have achieved major molecular response with nilotinib.^51^ The ongoing phase 3 ENESTcmr trial is comparing the kinetics of complete molecular response to nilotinib versus imatinib, and effects on survival, among patients with persistent disease despite ≥2 years of imatinib therapy.^53^ Also, a phase 4 single-arm study (SENSOR) is studying the major molecular response rate after 12 months of nilotinib therapy in patients with suboptimal response to imatinib at 18 months.^54^

### Treatment with dasatinib in patients resistant or intolerant to imatinib

Treatment with dasatinib was studied in an open-label, randomized, phase 3, dose-optimization trial conducted in patients with CML CP who were imatinib-resistant or imatinib-intolerant. Patients were randomly assigned to 1 of 4 dasatinib treatment groups: 100 mg once daily (n = 167), 50 mg twice daily (n = 168), 140 mg once daily (n = 167), or 70 mg twice daily (n = 168).^55^ Although the results were not stratified according to suboptimal response at study initiation, 43% of patients entered the study with CHR, and 17% of patients entered with major cytogenetic response. Results at 48 months of follow-up were recently reported.^56^ Response rates were similar across all 4 treatment arms, and PFS was similar in patients with or without baseline *BCR-ABL* mutations. The authors concluded that dasatinib 100 mg once daily has an acceptable benefit-risk profile in patients failing or responding suboptimally to imatinib.

In addition, a phase 2-3 open-label study of dasatinib versus imatinib 800 mg daily in patients with a suboptimal response to imatinib was recently completed, and a prospective, open-label, randomized study evaluating the efficacy of dasatinib and imatinib 600 mg daily is ongoing in Japan. These prospective studies will better define the efficacy and safety of different treatment options, including escalated imatinib doses and treatment with nilotinib and dasatinib, in patients with a suboptimal response to imatinib.

With the recent regulatory approvals and availability of positive, longer-term data for front-line nilotinib and dasatinib therapy in CML patients,^57^,^58^ more clinicians will likely prescribe these drugs for newly diagnosed patients. There are no clinical data yet describing suboptimal response to first-line therapy with nilotinib or dasatinib. As experience with these agents in newly diagnosed patients accumulates, the definitions of suboptimal response and failure may need to be revisited. Some clinicians may be concerned that patients with suboptimal responses to nilotinib or dasatinib may have poorer outcomes than patients with similar responses to imatinib; the prognosis of these patients will become clear with greater clinical experience. Furthermore, the best way to manage these patients is not clear. The outcomes after switching from first-line nilotinib to dasatinib (and vice versa) or changing to imatinib, or a clinical trial of an experimental agent (eg, bosutinib or ponatinib) remain to be studied. Although data on response to second-line nilotinib after first-line dasatinib failure or dasatinib response after first-line nilotinib failure have not yet been reported, nilotinib has shown favorable third-line response in a single phase 2 study in patients who failed imatinib and dasatinib.^59^ Similarly, third-line response to dasatinib has been demonstrated in a small study of patients who failed both imatinib and nilotinib.^60^

### Treatment with an investigational agent

Patients not achieving optimal response to currently available treatments can be considered for a clinical trial of an investigational agent.^17^ Several targeted therapies are in clinical development. Preliminary results from an ongoing phase 1 dose-escalation study of the multikinase, pan–BCR-ABL inhibitor ponatinib (AP24534) showed antileukemic activity in patients with refractory CML, including patients with the T315I mutation.^61^ Phase 1 and 2 trials of ponatinib are ongoing in patients with refractory CML and acute lymphoblastic leukemia. Danusertib (PHA-739358) is a multikinase aurora inhibitor with in vitro activity against wild-type ABL and ABL/T315I^62^; preliminary results from an ongoing phase 1 dose-escalation study indicate antileukemic activity in patients with AP or BC who failed imatinib and/or nilotinib or dasatinib.

Bosutinib (SKI-606), a dual Src/ABL inhibitor, is the furthest along in clinical development for CML. Preliminary results from a phase 1-2 study in patients in CP with resistance or intolerance to imatinib demonstrated bosutinib efficacy in patients failing to achieve optimal response to imatinib.^63^ Bosutinib had a favorable toxicity profile in both phases of the study. Bosutinib has also been studied in the third-line setting. An open-label phase 1-2 study of patients who failed imatinib therapy and were resistant or intolerant to dasatinib, or resistant to nilotinib, demonstrated that 13% of patients achieved complete cytogenetic response and 26% achieved major cytogenetic response at 6 months of bosutinib therapy. Bosutinib was well tolerated in these patients, although 27% of dasatinib-intolerant, 14% of dasatinib-resistant, and 11% of nilotinib-resistant patients discontinued treatment because of AEs.^64^

## Conclusions

Since the discovery of the Ph chromosome 50 years ago,^65^ management of CML has evolved significantly. Identification of the oncogenic fusion protein BCR-ABL producing a constitutively active tyrosine kinase^4^ led to the rational development of TKIs. In the landmark IRIS trial, imatinib demonstrated significantly improved long-term outcomes compared with an IFN-alpha/cytarabine combination regimen.^10^ With the more potent TKIs, nilotinib and dasatinib, the need to quantify responses beyond cytogenetic responses became apparent. Therefore, alongside the development of more effective therapies, more sensitive monitoring techniques are becoming a standard of care in the management of CML.

The European LeukemiaNet and National Comprehensive Cancer Network guidelines are evolving as experience with TKIs grows and clinical data mature. As the predictive value of cytogenetic and molecular responses at given time points becomes clearer, physicians are better able to identify patients at risk of failure or suboptimal response. Patients who fail first-line therapy have unequivocally poorer outcomes compared with optimal responders.^25^,^26^ Patients with early suboptimal responses tend to have poor long-term outcomes, and a change in treatment should be considered for these patients. The best treatment approach for these patients is still an active area of research.

There are 3 key issues to consider in patients with suboptimal response: 1) identification of suboptimal response, which requires familiarity with the milestone responses typically achieved during imatinib treatment at specific intervals; 2) monitoring frequency, particularly for cytogenetic analysis and molecular monitoring of *BCR-ABL* transcript levels (it is noteworthy that according to 1 survey, not all physicians perform cytogenetic analysis or polymerase chain reaction testing according to guideline recommendations^66^); and 3) treatment of suboptimal response, which requires regular clinical assessment. Physicians may be hesitant to switch to a second-line therapy when the first-line treatment continues to provide benefit.^9^

Despite incremental short-term and medium-term gains associated with continued imatinib therapy—even at higher doses—in patients with suboptimal responses, the long-term prognosis with this strategy remains poor. Preliminary data suggest that switching to second-line therapies may be effective for patients with suboptimal response, but this needs confirmation.^46^,^67^-^72^ Comparison of the relative efficacy of imatinib dose escalation and next-generation TKIs awaits results from prospective randomized studies. This is an exciting era in CML management. With more effective treatments, increasingly sensitive monitoring techniques, and the ability to identify patients at risk of suboptimal response and poor long-term outcomes, physicians move closer to individualized care of patients for longer patient survival.

## FUNDING SOURCES

Financial support for medical editorial assistance was provided by Novartis Pharmaceuticals Corporation.

## CONFLICT OF INTEREST DISCLOSURES

The authors made no disclosures.
